# Early Developmental Changes of Muscle Acetylcholine Receptors Are Little Influenced by Dystrophin Absence in *mdx* Mouse

**DOI:** 10.3390/life12111861

**Published:** 2022-11-12

**Authors:** Marta Morotti, Alessandro Gaeta, Cristina Limatola, Myriam Catalano, Maria Amalia Di Castro, Francesca Grassi

**Affiliations:** 1 Department of Physiology and Pharmacology, Sapienza University of Rome, 00185 Rome, Italy; 2 Laboratory Affiliated to Istituto Pasteur Italia, Department of Physiology and Pharmacology, Sapienza University of Rome, 00185 Rome, Italy; 3 Istituto di Ricovero e Cura a Carattere Scientifico (IRCCS) Neuromed, 86077 Pozzilli, Italy

**Keywords:** Duchenne muscular dystrophy, neuromuscular junction, acetylcholine receptors, dystrophic muscle, *mdx*, receptor clustering, patch clamp

## Abstract

Dystrophin is a cytoskeletal protein contributing to the organization of the neuromuscular junction. In Duchenne muscular dystrophy, due to dystrophin absence, the distribution of endplate acetylcholine receptors (AChRs) becomes disorganized. It is still debated whether this is due to the absence of dystrophin or to the repeated damage/regeneration cycles typical of dystrophic muscle. We addressed this controversy studying the endplate in the first 3 postnatal weeks, when muscle damage in dystrophic (*mdx*) mice is minimal. By synaptic and extra-synaptic patch-clamp recordings in acutely dissociated *mdx* and wt muscle fibers, we recorded unitary events due to openings of AChR-channels containing the γ and ε subunit. We also examined AChR distribution at the endplate by immunofluorescence assays. No differences between wt and *mdx* fibers were found in the γ/ε switch, nor in the AChR organization at the endplates up to 21 postnatal days. Conversely, we detected a delayed appearance and disappearance of patches with high channel opening frequency in *mdx* fibers. Our data emphasize that the innervation-dependent γ/ε switch and AChR organization in the endplate are not affected by the absence of dystrophin, while extra-synaptic AChR cluster formation and disassembly could be differentially regulated in *mdx* mice.

## 1. Introduction

Duchenne muscular dystrophy (DMD) is characterized by the absence of dystrophin, a cytoskeletal protein that organizes dystrophin-glycoprotein complexes (DGCs) in several tissues, with a prominent role in skeletal and cardiac muscle. In skeletal muscle, DGC bridges cytoskeletal actin filaments and the extracellular matrix and stabilizes sarcolemma during muscle contraction, preventing mechanical damage (reviewed in [1]). Dystrophin is also present at the muscle endplate, in particular in the depths of the junctional folds, where it binds voltage-gated Na^+^ channels and determines the length of synaptic folds [2]. At the crests, where acetylcholine receptors (AChR) are located, the paralogue utrophin is present [3,4]. Altogether, the DGC is crucial for maintaining the proper organization of the neuromuscular junction (NMJ). Indeed, morphological, and functional abnormalities of the NMJ are observed in DMD patients and dystrophic animals. From a structural point of view, denervation, partial overlap of pre- and post-synaptic structures and loss or reduced length of junctional folds have been observed in DMD patients [5], together with features suggestive of post-synaptic fragmentation [6]. The endplate shows a frankly fragmented structure in dystrophin-deficient *mdx* mice and in Golden Retriever GRMD dogs [7], accompanied by axonal sprouting in *mdx* mice but apparently not in dogs [7,8]. Functional changes are also observed [9], consisting in decreased postsynaptic response to acetylcholine (ACh) and a compensatory increase in quantal content [10]. Apparently, this is a clear example of synaptic homeostasis, well described for myasthenia gravis: a reduced postsynaptic response due to end-plate damage triggers retrograde processes that enhance neurotransmitter release [11].

Damage to the NMJ can impair neuromuscular transmission and contribute to weakness of dystrophin-lacking muscles, exacerbating disease progression [12]. Thus, it is important to understand how it occurs and whether it can be prevented. To date, it is still debated whether the damage is due to the absence of dystrophin or to the repeated cycles of damage and regeneration typical of dystrophic muscle fibers (see [13] vs. [7] for recent contributions on the two opposing views). Looking at NMJ development, which occurs in the first 3 weeks of life, before massive muscle damage, can yield clues to solve this uncertainty.

A key event in NMJ development is the loss of poly-neuronal innervation, which was found to be accelerated in the sternomastoid muscle of *mdx* mice at postnatal day 1 to 7 (P1 to P7), as compared to wild type (wt) animals [14]. Of note, up to 10–11 days of age, a negligible proportion of muscle fibers is undergoing, or recovering from, damage, and the ultrastructure of the endplate is still extremely simple and not overtly altered [15,16]. Since the loss of poly-neuronal innervation is due to a competition among the axons targeting the same muscle fiber [17], these data taken together suggest a differential synaptic function from the earliest stages of NMJ development in *mdx* vs. wt mice, independent of structural damage.

To test whether this is indeed the case, we looked for an independent indicator of NMJ activity. Nerve-muscle contact and the ensuing electrical activity cause change in subunit composition of acetylcholine receptors (AChR) and in their localization over the sarcolemma [17]. Nerve-released agrin triggers the expression of the gene coding the ε subunit in sub-synaptic nuclei, while muscle depolarization represses the expression of genes coding AChR subunits in extra-synaptic nuclei. Therefore, in non-innervated muscles, there is a widespread presence of receptors formed by 2α_1_βγδ subunits (γ-AChR), while AChRs formed by 2α_1_βεδ subunits (ε-AChR) are present in innervated NMJs (as reviewed in [18]). In the rat, AChRs disappear from the extra-junctional sarcolemma with a time course that parallels the loss of poly-neuronal innervation [19].

We reasoned that, if NMJ activity is increased from early stages in *mdx* mice, AChR dynamics would be affected. Thus, we studied ACh-induced unitary channel openings in synaptic and non-synaptic membrane of acutely dissociated fibers from the flexor digitorum brevis muscle of *mdx* and wt muscles between P7 and P21, when salient milestones of synaptic maturation are achieved. In this muscle, the percentage of fibers that undergo degeneration/regeneration cycles is particularly low [20] so that any observed difference would be due to the absence of dystrophin. At the same time points, we examined the appearance of the endplate in tibialis anterior muscle, a widely used muscle in studies on *mdx* mice. At no time did we detect structural or functional differences between wt and *mdx* fibers, suggesting that (absence of) dystrophin has little effects on the early steps of NMJ development in hindlimb muscles.

## 2. Materials and Methods

### 2.1. Animals

C57BL/10ScSn (wild type, wt) and C57BL/10ScSn-Dmd<*mdx*>/J (*mdx*) mice, purchased from Jackson Laboratory, were bred in our facility, housed in standard cages at a constant temperature (22 ± 1 °C) and relative humidity (50%), with a 12:12 h light: dark cycle (light on 07.00–19.00 h). Food and water were available ad libitum. Experiments were authorized by the Italian Ministry of Health (Authorization n. 320/2020).

### 2.2. Muscle Fiber Preparation

Flexor digitorum brevis (FDB) and tibialis anterior muscles were dissected from the hindlimbs of male and female mice aged 7 to 21 days, sacrificed by cervical dislocation. The FDB muscles were washed in PBS and incubated with Type I collagenase (3 mg/mL, Sigma) for 30 min at 37 °C in Minimum Essential Medium (MEM, Gibco, Thermo Fisher Scientific, Waltham, MA, USA, #11095-080). After equilibrating in Ca^2+^-free MEM (Gibco, Thermo Fisher Scientific, Waltham, MA, USA, #11380-037) for 30 min at room temperature, muscles were gently triturated using plastic Pasteur pipettes to obtain single fibers. The fibers were then transferred to 35 mm Petri dishes and equilibrated in a Ca^2+^-free solution (see below) for recording.

### 2.3. Cell-Attached Recordings

Recordings were performed on fibers imaged by a phase-contrast microscope (Axioskop 2 FS, Zeiss, Jena, Germany). Patch pipettes were filled with a solution containing (in mM, all from Sigma Aldrich, St. Louis, MO, USA): NaCl 140, KCl 2.8, CaCl_2_ 2, MgCl_2_ 2, glucose 10, HEPES/NaOH 10, pH 7.3, plus ACh (200 nM). The resistance was 4–6 MΩ. The fiber bathing solution had the same composition, except for the omission of CaCl_2_. Single-channel currents were recorded at room temperature (23–26 °C) using low-noise Axo-patch 200 B amplifier (Molecular Devices, San Jose, CA, USA) in cell-attached mode. Data were sampled at 25 kHz, digitally filtered at 5 kHz (Gaussian filter) and analyzed using pClamp 9.2 (Molecular Device, San Jose, CA, USA). Only single openings (100–3000 at each pipette potential in every patch) were used to obtain the channel slope conductance and duration. Measurements were performed at the same pipette potential at the beginning and at the end of each recording, to check that membrane potential remained stable. Slope conductance was calculated only if membrane potential was stable and unitary events were recorded at least at 3 different pipette potentials. When γ- and ε-AChR-channel populations were present together, the distribution of channel amplitude showed 2 gaussian components. The distribution of channel open times was analyzed only if the amplitude of γ- or ε-AChR channels were well separated. The kinetic properties of ACh-evoked events were compared at an estimated membrane potential of −90 ± 10 mV, calculated assuming a reversal potential of 0 mV for both γ- and ε-AChR-channels. Histograms of open times were fitted by a non-linear algorithm (pClamp 9.2, Molecular Device, San Jose, CA, USA) with the sums of exponential components, as required. Time constants are indicated as τ_op_. Total channel opening frequency was evaluated in each patch as (total number of events detected)/(total duration of the recording).

### 2.4. Endplate Evaluation

To evaluate the shape of the endplate, tibialis anterior muscles were fixed in 4% paraformaldehyde and snap frozen in isopentane at −80 °C. Longitudinal cryostat sections (20 μm) were incubated with rhodamine-conjugated alpha-bungarotoxin (α-BTX; Molecular Probes, Thermo Fisher Scientific, Waltham, MA, USA; B13423; 6 mg/mL) in DMEM plus 20% fetal bovine serum (FBS; Sigma, St. Louis, MO, USA) for 50 min at 37 °C. Thereafter, slices were extensively washed with the same medium, then with PBS. Images of AChRs stained by α-BTX were obtained using a system integrated by Crisel Instruments (Rome, Italy), comprising a CoolSNAP camera (Photometrics, Tucson, AZ, USA) coupled to an ECLIPSE Ti-S microscope (Nikon, Tokio Japan) and processed using MetaMorph 7.6.5.0 image analysis software (Molecular Device, San Jose, CA, USA), after background subtraction. A deconvolution algorithm was applied on z-stacked images. Junctions were defined as *continuous* if AChRs formed a continuous ribbon with 0–2 interruptions, or *fragmented* if 3 or more AChR clusters were present [13]. 50 to 110 endplates per mouse were scored.

### 2.5. Statistics

Data are presented as mean ± standard deviation. Statistical significance was analyzed using the non-parametric Mann–Whitney U-test for comparisons between two groups, the Kruskal–Wallis test for 3 or more groups.

## 3. Results

### 3.1. ACh-Evoked Channel Openings

To determine whether the timing of innervation and the response of muscle fibers to nerve-induced changes was altered in *mdx* muscle as compared to wt, the first aspect to analyze was the appearance of ε-AChRs and the disappearance of γ-AChRs. At P7 and P11, patch-clamp recordings were performed at random locations on muscle fibers, sampling the extra-synaptic membrane. Starting from P14, the endplate could be recognized as a protrusion of the membrane in most fibers, as previously reported [21]. Thus, at P14 and P21, recordings on the synaptic region (Figure S1) were done, in addition to extra-synaptic recordings performed at random locations. Overall, more than 150 fibers were analyzed.

**Figure 1:**
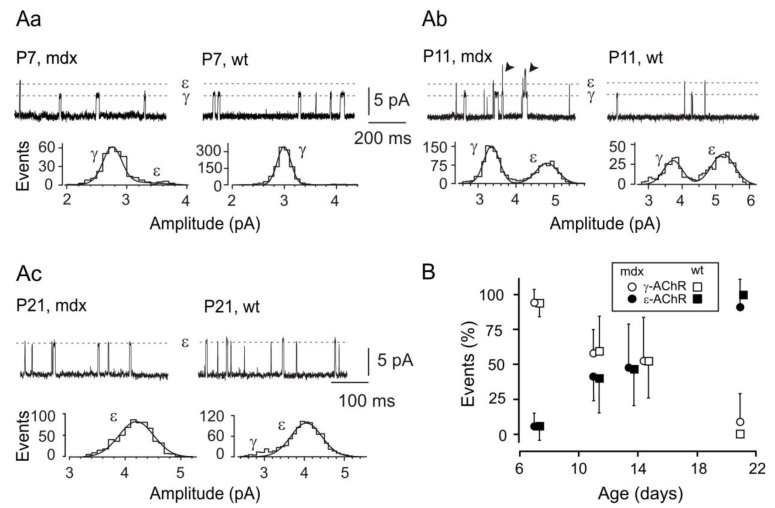
The expression of the γ and ε AChR subunits has similar time course in wt and *mdx* fibers. (**Aa**–**Ac**) typical ACh-evoked unitary events recorded in fibers from mice of the indicated strain and ages (top rows). Dashed grey lines indicate the amplitude of γ- and ε-AChR channel openings. For each recording, the distribution of γ- and ε-AChR channel amplitude is illustrated (bottom rows). (**Aa**,**Ab**), extra-synaptic patches; in (**Ab**), arrowheads indicate superimposed channel openings. (**Ac**): recording on the synaptic region. All traces were recorded at potentials (pipette potential + membrane potential) of −110 to −125 mV. (**B**) Percentage of events due to openings of γ- (open symbols) and ε-AChR channels (closed symbols) in *mdx* (circles) and wt (squares) fibers. At P14 and P21, only data for extra-synaptic patches are shown. Symbols have been displaced along the horizontal axis for clarity. At each age, values for wt and mdx fibers were not significantly different (*p* > 0.7).

In both wt and *mdx* fibers, at P7 only openings of γ-AChR channels were detected in most patches, with few events attributable to ε-AChRs detected in a minority of recordings (Figure 1Aa,B). Overall, openings of γ-AChR channels represented about 95% of all recorded events (Table S1). At P11 and P14, the two channel types contributed almost equally to unitary activity (Figure 1Ab,B, Table S1). Finally, at P21 events due to openings of ε-AChR channels were recorded in all patches, while γ-AChR channel activity was detected in few patches (Figure 1Ac and Table S1). In synaptic patches at P14 and P21, openings of γ- and ε-AChRs occurred with frequencies similar between wt and *mdx* fibers (Table S1). In most recordings, overlapping openings of two or more channels, were present. Together, these data indicate that the expression of the γ and ε AChR subunits has a remarkably similar timing in wt and *mdx* fibers.

For both γ-and ε-AChR channels, slope conductance was similar in *mdx* and wt mice at all ages (Table 1). On average, there were only minimal differences between synaptic and extra-synaptic events at P14 and P21 (Table 1).

**Figure 2:**
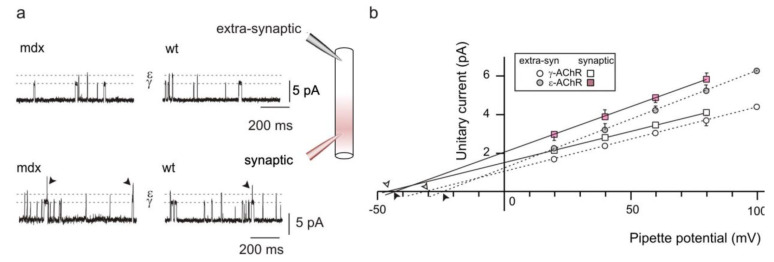
Unitary events in synaptic and extra-synaptic patches are very similar. (**a**) typical events recorded in *mdx* and wt fibers (P14) patched on the endplate (bottom traces, pink pipette in the inset) and then on the extra-synaptic membrane (top traces, grey pipette). All traces were recorded at potentials (pipette potential + membrane potential) of −110 to −125 mV. Dashed grey lines indicate the amplitude of γ- and ε-AChR channel openings. Arrowheads mark superimposed channel openings. (**b**) Slope conductance of γ- and ε-AChR channels in a different *mdx* fiber (P14) patched at the endplate (squares) and then in the extra-synaptic membrane (circles). Conductance values were 32.8 pS and 47.9 pS (synaptic, γ- and ε-AChR channels, respectively); 33.5 and 50.3 pS (extra-synaptic). Arrowheads indicate the reversal potentials, depolarized by about 20 mV in the second recording on the same fiber.

Several fibers were patched twice, first at the endplate and afterwards at an extra-synaptic location approximately midway between the endplate and the myotendineous junction. In all instances, slope conductances were similar, although membrane potential was often depolarized in the second recording (Figure 2), likely due to the stress of pipette withdrawal.

**Figure 3:**
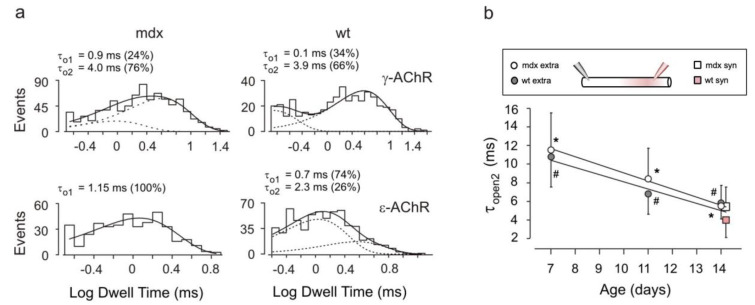
Open channel duration. (**a**) Typical distributions of open durations for γ-AChR channels (top) were fitted by the sum (solid line) of two exponential components (dotted lines) with the indicated time constants and relative weight. For ε-AChR channels (bottom), either a single (left) or two (right) exponential components were required. Synaptic recordings performed at P14 on one *mdx* and one wt fiber, as indicated. Membrane potential, −100 for all panels. (**b**) The time constant of the longer exponential component of γ-AChR channel openings significantly decreases with age in *mdx* (open circles) and wt (grey circles) fibers in extra-synaptic patches (*: *p* = 0.0002 for mdx, #: *p* = 0.001 for wt, Kruskal–Wallis test). At P14, values for τ_op2_ were not significantly different in synaptic (squares) and extra-synaptic patches (circles; *p* > 0.1). Symbols have been displaced along the horizontal axis for clarity.

The duration of channel openings also showed little differences between wt and *mdx* fibers of the same age. For γ-AChR channels, open durations were distributed as the sum of two exponential components and the longer time constant (τ_op2_) significantly decreased from P7 to P14 (Figure 3 and Table S2) in both strains. At P21, events attributable to γ-AChR channels were too rare for an adequate analysis.

For ε-AChR channels, the open time distribution could be fitted to a single exponential component in over 90% of the patches, with similar time constants for *mdx* and wt fibers. In the few remaining recordings, occurrence of longer events required a second exponential component (Figure 3 and Table S2), again with no difference between *mdx* and wt animals.

**Figure 4:**
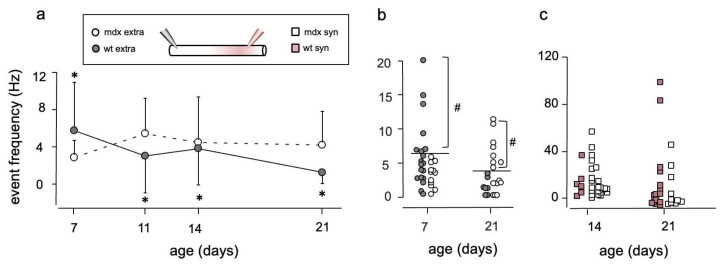
Frequency of channel openings changes with age. (**a**) In extra-synaptic patches on wt fibers (closed circles), total frequency of AChR channel openings decreases with age. In *mdx* fibers (open symbols), changes in event frequency are not significantly different. *: *p* = 0.015 by Kruskal–Wallis test. (**b**) Scatter plot of channel frequency in all recorded fibers at P7 and at P21. At P7, channel frequencies above 5.8 Hz (horizontal line) are found in 7/19 wt fibers, 0/12 *mdx* fibers. At P21, frequencies above 3.3 Hz occur in 8/15 *mdx* fibers, 0/ 7 wt fibers. #: *p* = 0.026 at P7, 0.022 at P21 by Fisher’s exact test. (**c**) In synaptic patches the frequency of events shows a large scattering in both wt (pink squares) and *mdx* (empty squares) fibers, at P14 and P21. In (**b**,**c**), symbols have been displaced along the horizontal axis for clarity.

Another point to consider is that electrical activity in muscle fibers causes the dispersal of non-synaptic AChR clusters and prevents expression of AChRs in the extra-synaptic membrane, so that channel density is expected to decrease over time. Conversely, in the synaptic region AChRs accumulate during endplate maturation. Assuming that channel open probability remains constant over time, the frequency of channel openings reflects the number of AChRs in the patch, which is proportional to channel density. In wt fibers, channel frequency in non-synaptic patches was maximal at P7, then significantly decreased (*p* = 0.015; Kruskal–Wallis test), in line with the accepted idea that extra-synaptic AChR density drops as muscle fiber matures. In *mdx* fibers, the differences among ages were not statistically significant (*p* = 0.33; Kruskal–Wallis test; Figure 4a).

In wt fibers, the range of variability also decreased with age, with fewer and fewer recordings showing a high frequency of events, indicating that regions of high channel density decreased during the P7–P21 period. In particular, 13 out of 19 (13/19) recordings performed at P7 had opening frequency higher than any recording obtained at P21 (*p* = 0.005; Fisher exact test; Figure 4b). Conversely, in *mdx* fibers recordings with high activity were present at all ages, with no significant difference between P7 and P21 (*p* = 0.1 Fisher exact test, Figure 4b).

When comparing wt and *mdx* fibers, at P7 the percentage of high-frequency recordings was significantly lower in *mdx* than in wt fibers (*p* = 0.026). Conversely, at P21, 50% of the recordings in *mdx* fibers had higher frequency of openings than any recording in wt fibers (*p* = 0.022; Fisher exact test). Together, these results are suggestive of a slowed reduction of the density of extra-synaptic AChRs in *mdx* mice.

In synaptic patches, the frequency of events was significantly higher than in extra-synaptic recordings (*p* < 0.04), and showed a large variability in fibers of both strains (Figure 4c).

### 3.2. Endplate Structure

**Figure 5:**
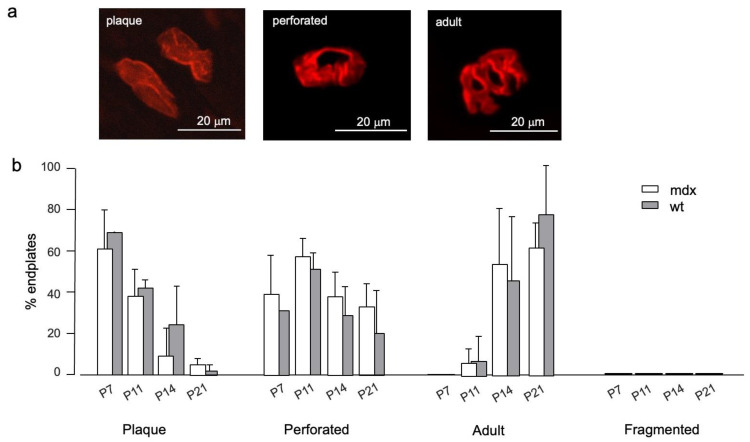
AChR organization at endplates. (**a**) Examples of the different endplate morphology in the tibialis anterior of a P14 *mdx* mouse, as revealed by staining AChRs with a-BuTX. (**b**) Percentage of the different endplate types at the indicated ages. Error bars indicate SD. Values for wt and mdx fibers were not significantly different (*p* > 0.3). Notice the absence of fragmented endplates at all ages.

We also assessed the development of the NMJ examining AChR distribution at endplates in the tibialis anterior muscle, well characterized in muscular dystrophy, using rhodamine-conjugated alpha-bungarotoxin to visualize AChRs (Figure 5). At P7, most endplates presented the immature plaque-like shape, although some had progressed to a perforated appearance, in both *mdx* and wt mice. Endplates with an adult-like morphology were first detected at P11, and their percentage increased with age, while the more immature forms became less and less represented (Figure 5). No fragmented endplates were observed, even at P21, indicating that damage does not occur in this early period.

## 4. Discussion

In this study we have investigated the time course of the changes in the expression of AChR subunits imparted by NMJ maturation in *mdx* and wt FDB muscle fibers in a time window which has received little attention before. We show that the relative abundance of γ- and ε-AChR channel openings changes with remarkably similar time courses in *mdx* and wt fibers, both in the extra-synaptic and in synaptic regions. By contrast, the total frequency of γ- and ε-AChR channel openings, an indicator of channel density, in extra-synaptic patches was differentially regulated in the two strains. In wt fibers, the range of variability decreased from P7 to P21, while it increased in *mdx* fibers.

Reportedly, FDB muscle undergoes limited structural damage in *mdx* mice [20], and the enzymatic digestion procedure used for fiber isolation further ensures that only healthy, non-damaged fibers were considered in this study. Thus, we are confident that we have selectively investigated the consequences of dystrophin absence, without contamination by muscle damage.

Studies in rat diaphragm and soleus show that AChRs are present on the whole fiber in the first postnatal days [22], then they progressively become concentrated at the endplate over the course of the first 3 postnatal weeks, while channels with high conductance and brief open duration (ε-AChR) replace channels with low conductance and long openings (γ-AChR; [23]). Muscle denervation prevents the conversion [24]. Thus, the gradual appearance of the ε subunit and the concomitant disappearance of the γ provide a functional readout of effective nerve-muscle interaction and the patch clamp technique offers a very sensitive assay of the presence of AChRs containing either subunit in the sarcolemma of muscle fibers. Differences in the time course of γ-to-ε switch would be expected under the hypothesis that the faster loss of poly-neuronal innervation observed in the sternomastoid muscle [14] is due to an accelerated development of the NMJ.

In this work, we analyzed a large number of FDB muscle fibers (over 85 *mdx* and 70 wt) from mice aged between P7 and P21, when the NMJ is considered to achieve maturity. ACh-evoked channel openings were detected in all the synaptic recordings and in 75–100% of the extra-synaptic recordings performed. At all ages, unitary events could be ascribed to openings of γ- and ε-AChR channels based on their slope conductance and open duration. Both functional parameters were quite similar in *mdx* and wt fibers, and had the values expected from the literature [25,26]. The only unexpected finding was the decrease of the open duration of γ-AChR channels between P7 and P14, with a parallel time course in *mdx* and wt mice. A similar phenomenon has been observed at earlier ages in Balb/C mice and attributed to a developmentally regulated expression of a splice variant of the γ subunit [27]. The data obtained in the present study are compatible with the idea that the alternative splicing of the γ subunit occurs with a strain-specific timing and is unaffected by the absence of dystrophin. However, we cannot exclude subtle differences in the activation steps in the AChRs receptor in the mdx mice [28].

Our results show that the relative abundance of γ- and ε-AChR channel openings changes with remarkably similar time courses in *mdx* and wt fibers, both in the extra-synaptic and in synaptic regions. Thus, we found no functional indication of a differential regulation of the nerve-induced expression of the ε subunit and in the activity-dependent suppression of the γ subunit.

By contrast, the total frequency of γ- and ε-AChR channel openings in extra-synaptic patches, but not in synaptic ones, was differentially regulated. This parameter represents a reasonable indicator of channel density: the more AChRs are present in the patched membrane, the more likely their opening is. In wt fibers, opening frequency decreased from P7 to P21, while in *mdx* fibers it was fairly constant. Furthermore, recordings with high frequency of channel openings occurred in wt more than in *mdx* fibers at P7, the contrary at P21. This difference can be due to a differential regulation of the accumulation and dispersion of prepatterned (spontaneous) AChR aggregates. In vitro, AChR aggregates appear smaller in *mdx* than wt myotubes [29], but our data in vivo are suggestive of a slowed rather than impaired formation of large AChR clusters. At later ages, ACh-evoked ion influx causes dispersal of pre-existing AChR clusters through the action of intracellular intermediates, in particular cyclin-dependent kinase 5 (Cdk5) and calpain [30,31,32]. It tempting to speculate that (some of) the relevant signaling pathways are compromised in the absence of dystrophin.

In synaptic patches at both P14 and P21, recordings with high channel frequency were equally common in wt and *mdx* mice, again suggesting that innervation-related events have a similar time course. The frequency of channel openings showed a large scattering in both *mdx* and wt fibers, possibly, due to the uneven AChR distribution in endplates, which have a perforated or pretzel-like appearance in most endplates at both P14 and P21. Patches performed on the “holes” correspond to recordings with low frequency of events, while very high frequencies occurred when regions with high AChR density were patched.

Of note, the distribution of AChRs at the endplate progressed with minor, non-significant differences in the tibialis anterior of *mdx* and wt mice, and no sign of endplate fragmentation was observed at P14 and P21. These observations contrast with findings in the sternomastoid muscle of *mdx* mice, where endplate fragmentation was detected already at P14 [14]. Possibly, the craniosacral pattern of muscle development [33] contributes to this difference: the slow development of hindlimb muscles (common to FDB and tibialis anterior) protects them from damage, and hence from endplate fragmentation. It must be noted that our analysis was not aimed at describing the fine details of NMJ structure. Further morphological studies using super-resolution imaging techniques will disclose the detailed organization of AChRs distribution in the junctional folds [34].

A novel finding of this study is that, in *mdx* and wt fibers alike, openings of γ- and ε-AChR channels were detected in the extra-synaptic membrane at P11 and P14 and occasionally even at P21, time points that have received little attention before. In cell-attached patches on adult *mdx* (but not wt) fibers, a mixture of γ- and ε-AChR channel openings has been described and attributed to cycles of muscle degeneration/denervation [35]. In the extra-synaptic portion of wt adult FDB fibers, rare ε-AChR-channel openings and occasional openings of γ-AChR-channels have been described [21], but not confirmed in other studies [35]. In developing wt endplates, a mixture of γ- and ε-AChR is expressed at the endplate [25,26]. We show that openings of both channel subtypes are detected also in the extra-synaptic membrane in young animals, suggesting that during NMJ maturation both AChR isoforms are present in the extra-synaptic region, with the same abundance found at the synapse. This is at odds with the established idea that nerve-released agrin selectively induces the expression of ε subunit in subsynaptic nuclei [17,36]. In multinucleated muscle fibers, mRNA localization is necessary to maintain distinct compartments in the fiber. Most mRNAs accumulate close to the nucleus of origin, with only few large transcript diffusing away through interactions with microtubules [37]. In particular, in young adult mice, AChR-subunit mRNAs are at least 100 times more abundant in the junctional than in the extra-junctional region of the fiber. However, subsynaptic nuclei start to accumulate between P7 and P14 in wt mice [38], so it is tempting to speculate that mRNA confinement processes become fully operative during the third postnatal week, effectively restricting the presence of AChR channels to the synaptic membrane only once muscles reach maturity.

Altogether, our findings show that, although in non-damaged FDB muscle fibers, events dependent on the function of the neuromuscular synapse, such as the γ / ε-AChR switch, are not affected by the absence of dystrophin, the timing of clustering of AChRs could be slowed in *mdx* mice. Further studies are warranted to investigate more in detail the AChR organization at the endplate [34] or the kinetics of AChR-channel openings in the early development of mdx mice [28].

## Figures and Tables

**Table 1:** Slope conductance of ACh-evoked channel openings.

	*mdx*		wt	
Age (Days)	γ-AChR (pS)	ε-AChR (pS)	γ-AChR (pS)	ε-AChR (pS)
7	30.8 ± 2.6 (10)	46.1 ± 3.4 (4)	33.3 ± 2.8 (17)	47.1 ± 4.0 (4)
11	33.6 ± 3.0 (20)	48.6 ± 4.7 (21)	33.0 ± 3.0 (19)	48.9 ± 5.8 (18)
14 extra	34.8 ± 2.7 (19)	50.2 ± 3.7 (19)	34.9 ± 4.2 (8)	52.8 ± 6.6 (9)
14 syn	33.8 ± 3.2 (18)	48.9 ± 5.1 (19)	35.9 ± 4.3 (6)	52.6 ± 5.2 (6)
21 extra	31.7 (2)	48.1 ± 4.2 (10)	n.d.	48.1 ± 5.9 (5)
21 syn	31.2 (2)	45.1 ± 5.4 (9)	34.8 (1)	49.6 ± 6.6 (15)

Values represent mean ± SD (number of patches). At P14 and P21, separate values are given for synaptic (syn) and extra-synaptic (extra) recordings. n.d.: not determined. For each AChR isoform, slope conductances of wt and *mdx* fibers were not statistically different (*p* > 0.1).

## Data Availability

Relevant data is contained within the article or Supplementary Material.

## Supplementary Materials

The following supporting information can be downloaded at: https://www.mdpi.com/article/10.3390/life12111861/s1, Figure S1: Patching the synaptic region; Table S1: Percentage of channel openings due to γ-AChR and ε-AChR; Table S2: Best fitting time constants of ACh-evoked open channel distributions.
