# Supplementary material for: Early Developmental Changes of Muscle Acetylcholine Receptors Are Little Influenced by Dystrophin Absence in mdx Mouse
Source: Life (Basel). 2022 Nov 12;12(11):1861. doi: 10.3390/life12111861 (PMC9696329; doi:10.3390/life12111861)
Supplement: Supplementary file 1 [file life-12-01861-s001.zip › life-2022305-supplementary.pdf]

Supplementary material

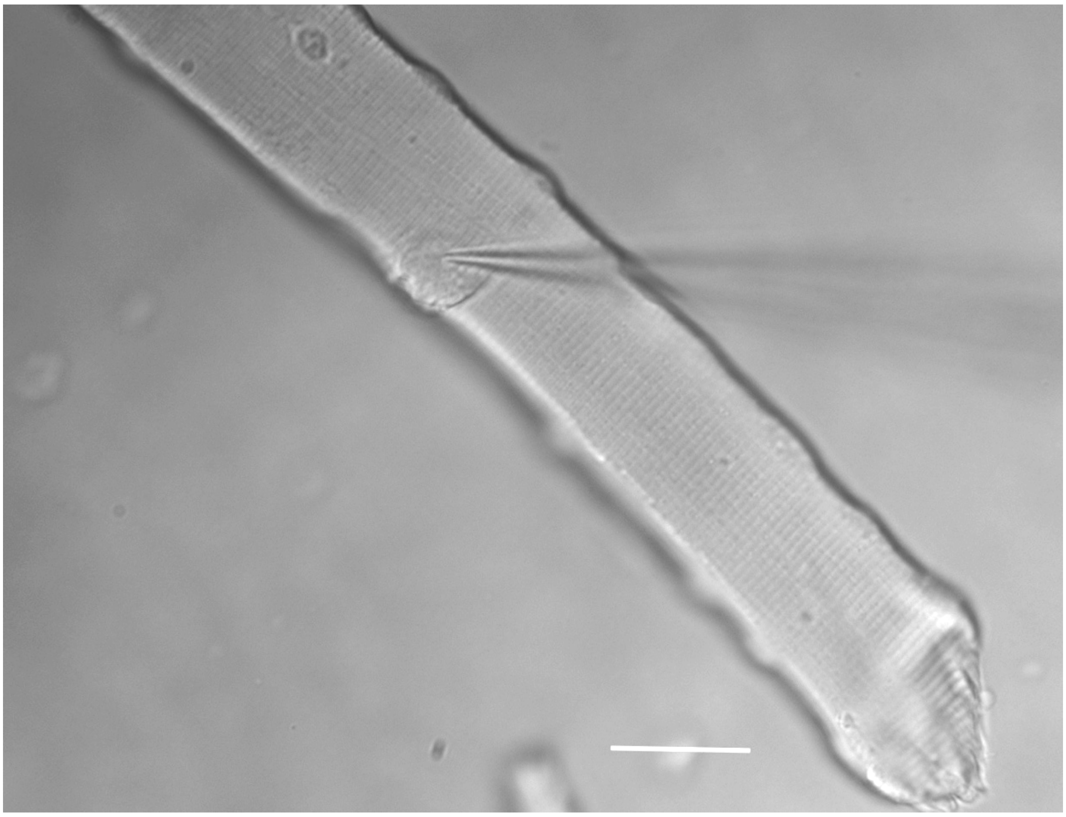

Figure S1. Patching the synaptic region. The endplate is visible as a rounded region in which striations are not visible. Patch pipette is on the left, only the tip is in focus. Scale bar, 20 µm.

Table S1. Percentage of channel openings due to γ-AChR and ε-AChR.

	mdx			wt		
age (days)	γ-AChR %	ε-AChR %	Detected in: γ; ε	γ-AChR %	ε-AChR %	Detected in: γ; ε
7	94.2±9.4 (12)	5.8±9.4	12; 4	94±10 (18)	6±10	18; 5
11	58±17 (25)	42±17	24;25	60±25 (23)	40±25	23; 22
14 extra	52±31(21)	48±31	21; 17	53±27 (9)	47±27	8; 9
14 syn	44±16 (23)	56±16	22; 23	49±13 (6)	51±13	6; 6
21 extra	6 (14)	94±17	2; 14	0 (5)	100	5; 5
21 syn	6 (13)	94±11	2; 13	2 (15)	97.7 ± 8.8	1; 15

Values represent mean ± S.D (n. of patches) of the percentage of total unitary events attributable to γ-AChR and ε-AChR channel openings in each patch, taken as 0 when the population was not present. For each mouse strain, the third column reports the number of patches where γ-AChR; ε-AChR channel openings were detected.

Table S2. Best fitting time constants of ACh-evoked open channel distributions.

		mdx		wt	
Age (days)		γ-AChR	ε-AChR	γ-AChR	ε-AChR
7	τ_{op1} (ms); w_1 (%)	0.72± 0.40; 34	1.53±0.65; 100	1.36 ± 0.76; 39	1.9 ± 0.5; 100
	τ_{op2} (ms); w_2 (%)	11.5 ±4.0; 66		10.8 ± 3.3; 61	
11	τ_{op1} (ms); w_1 (%)	0.65±0.25; 34	1.62±0.66; 96	0.66 ± 0.56; 38	1.3 ± 0.4; 97
	τ_{op2} (ms); w_2 (%)	8.4 ± 3.3; 65		6.7 ± 2.6; 62	
14 extra	τ_{op1} (ms); w_1 (%)	0.49 ± 0.19; 41	1.17±0.34; 100	0.51 ± 0.26; 41	1.25 ± 0.32; 100
	τ_{op2} (ms); w_2 (%)	5.5 ± 2.2; 59		5.8 ± 1.7; 59	
14 syn	τ_{op1} (ms); w_1 (%)	0.69 ± 0.33; 33	1.08 ± 0.35; 97	0.42 ± 0.10; 35	0.83 ± 0.32; 95
	τ_{op2} (ms); w_2 (%)	5.4 ± 2.1; 67		4.0 ± 1.9; 65	
21 extra	τ_{op1} (ms); w_1 (%)	---	1.06 ± 0.51; 98	---	0.83 ± 0.18; 100
21 syn	τ_{op1} (ms); w_1 (%)	---	0.92 ± 0.26; 87.4	---	1.04 ± 0.52; 91.4
	τ_{op2} (ms); w_2 (%)		3.06 ± 0.57; 12.6		2.0; 8.6

In each patch, open channel distribution of γ- and ε-AChR channels were fit to one or two exponential components, as required. Values represent mean ± S.D of the time constants; weight of each component (averaged over all patches). At P14 and P21, separate values are given for synaptic (syn) and extra-synaptic (extra) recordings.
